# Influence of Ammonium on Formation of Mineral-Associated Organic Carbon by an Ectomycorrhizal Fungus

**DOI:** 10.1128/AEM.03007-18

**Published:** 2019-05-02

**Authors:** Tao Wang, Zhaomo Tian, Anders Tunlid, Per Persson

**Affiliations:** a Department of Biology, Microbial Ecology Group, Lund University, Lund, Sweden; b Centre for Environmental and Climate Research, Lund University, Lund, Sweden; University of California, Davis

**Keywords:** ectomycorrhizal fungi, decomposition, organic matter-mineral interaction, soil organic matter

## Abstract

The interactions between dissolved organic matter (DOM) and mineral particles are critical for the stabilization of soil organic matter (SOM) in terrestrial ecosystems. The processing of DOM by ectomycorrhizal fungi contributes to the formation of mineral-stabilized SOM by two contrasting pathways: the extracellular transformation of DOM (*ex vivo* pathway) and the secretion of mineral-surface-reactive metabolites (*in vivo* pathway). In this study, we examined how changes in nitrogen (N) availability affected the formation of mineral-associated carbon (C) from these two pathways. DOM was extracted from forest soils. The processing of this DOM by the ectomycorrhizal fungus Paxillus involutus was examined in laboratory-scale studies with different levels of ammonium. At low levels of ammonium (i.e., under N-limited conditions), the DOM components were slightly oxidized, and fungal C metabolites with iron-reducing activity were secreted. Ammonium amendments decreased the amount of C metabolites, and no additional oxidation of the organic matter was detected. In contrast, the hydrolytic activity and the secretion of N-containing compounds increased, particularly when high levels of ammonium were added. Under these conditions, C, but not N, limited fungal growth. Although the overall production of mineral-associated organic C was not affected by ammonium concentrations, the observed shifts in the activities of the *ex vivo* and *in vivo* pathways affected the composition of organic matter adsorbed onto the mineral particles. Such changes will affect the properties of organic matter-mineral associations and, thus, ultimately, the stabilization of SOM.

**IMPORTANCE** Nitrogen (N) availability plays a critical role in the cycling and storage of soil organic matter (SOM). However, large uncertainties remain in predicting the net effect of N addition on soil organic carbon (C) storage due to the complex interactions between organic matter, microbial activity, and mineral particles that determine the formation of stable SOM. Here, we attempted to disentangle the effects of ammonium on these interactions in controlled microcosm experiments including the ectomycorrhizal fungus P.involutus and dissolved organic matter extracted from forest soils. Increased ammonium levels affected the fungal processing of the organic material as well as the secretion of extracellular metabolites. Although ammonium additions did not increase the net production of mineral-adsorbed C, changes in the decomposition and secretion pathways altered the composition of the adsorbed organic matter. These changes may influence the properties of the organic matter-mineral associations and, thus, the stabilization of SOM.

## Abstract (precis)

Nitrogen (N) availability plays a critical role in the cycling and storage of soil organic matter (SOM). However, large uncertainties remain in predicting the net effect of N addition on soil organic carbon (C) storage due to the complex interactions between organic matter, microbial activity, and mineral particles that determine the formation of stable SOM. Here, we attempted to disentangle the effects of ammonium on these interactions in controlled microcosm experiments including the ectomycorrhizal fungus P.involutus and dissolved organic matter extracted from forest soils. Increased ammonium levels affected the fungal processing of the organic material as well as the secretion of extracellular metabolites. Although ammonium additions did not increase the net production of mineral-adsorbed C, changes in the decomposition and secretion pathways altered the composition of the adsorbed organic matter. These changes may influence the properties of the organic matter-mineral associations and, thus, the stabilization of SOM.

## INTRODUCTION

Soil organic matter (SOM) stores the largest quantity of carbon (C) in terrestrial ecosystems (1, 2). Microorganisms can decompose most SOM, and this results in the release of CO_2_ into the atmosphere. The remaining fraction of SOM is stabilized against microbial decomposition over centuries to millennia (2, 3). Changes in the magnitude of this stable SOM pool determine whether soils can act as a sink or a source of atmosphere CO_2_ in response to environmental changes and have therefore stimulated extensive research efforts (2).

Despite this research, the mechanisms by which SOM is stabilized are not well understood. According to the classical view, SOM becomes stabilized via polymerization reactions that lead to the formation of recalcitrant humic substances (4, 5). However, the humification model has been increasingly questioned by findings indicating that stable SOM is not rich in humic polymers (6). Instead, it was recently proposed that SOM becomes protected from microbial decomposition by interacting with mineral particles and by being incorporated into aggregates (7). Microorganisms contribute to the formation of such protected SOM by processing compounds in the litter material into smaller molecules (i.e., depolymerization). This is accompanied by an increased degree of oxidation of the processed products, which increases their water solubility, and possibly also their reactivity toward mineral particles, and their propensity to form aggregates consisting of the assembly of discrete molecules (7). This emerging view highlights the critical importance of dissolved organic matter (DOM) in the formation of stable SOM. DOM is also considered the most reactive organic matter fraction in soils and is subject to continuous microbial processing (8, 9).

**FIG 1:**
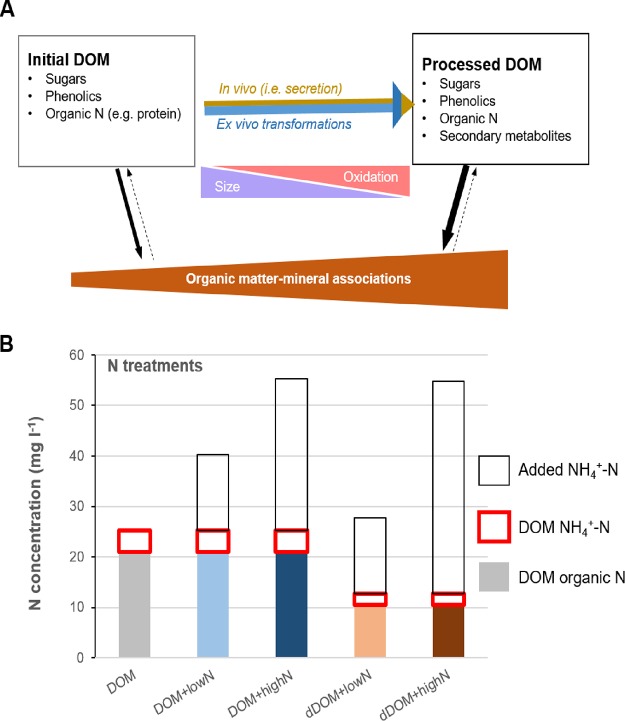
Conceptual model describing the formation of mineral-associated organic matter by fungi and the nitrogen treatments used in this study. (A) Fungal processing of dissolved organic matter (DOM) can contribute to the formation of mineral-associated organic C by two different pathways: extracellular transformation (*ex vivo*), including oxidation and depolymerization of compounds in the DOM, and the secretion of fungal metabolites (*in vivo*). Both modifications can enhance the retention of the processed DOM on mineral particles (10). The relative importance of each mechanism is indicated by solid-arrow thickness. Dotted arrows indicate the desorption process. (B) Nitrogen treatments used in this study.

Recent laboratory-scale experiments have shown that processing of DOM by both saprotrophic and ectomycorrhizal (ECM) fungi is able to enhance the formation of mineral-associated organic C, partly due to the depolymerization and oxidation of DOM (10). In addition, these fungi secrete substantial amounts (>10% new biomass C) of metabolites during DOM processing. Some of the metabolites are mineral-surface reactive and also contribute to the enhanced formation of mineral-associated organic C (10). These findings fit into a novel conceptual framework that emphasizes the involvement of microorganisms in stabilizing SOM (11). It is proposed that microorganisms influence SOM formation by two major pathways: (i) *ex vivo* transformation involving the action of extracellular enzymes that transform SOM into a material which is more stable with respect to microbial decomposition, and (ii) *in vivo* turnover mechanisms that, via the assimilation of organic matter-biosynthesis-growth-death, result in the release of stable microbially derived material (11). In the context of DOM, the secretion of fungal metabolites is a main component of the *in vivo* turnover pathway (Fig. 1).

Besides saprotrophic fungi, ECM fungi are the major functional group of fungi in boreal forest ecosystems (12). ECM fungi obtain their energy (i.e., glucose) from their host plants, and the mycelium is mainly located in deeper soil horizons that are enriched in decomposed and oxidized SOM (13). The ability of ECM fungi to decompose such SOM has been questioned due to the fact that these fungi have lost many genes encoding enzymes associated with plant litter decomposition that are present in saprotrophic fungi (14). However, recent studies have shown that ECM fungi have the capacity to decompose SOM using oxidative mechanisms (15–17). It has been suggested that these mechanisms are mainly used for mobilizing nutrients, including nitrogen (N), which are entrapped in complex SOM (18).

Soils in northern forest ecosystems have experienced an unintended ammonium (NH_4_^+^) fertilization due to N deposition, and the effects of N additions on SOM decomposition have been extensively studied (19, 20). Many of these studies have shown that N deposition suppresses microbial activity and thereby decreases SOM decomposition (19, 21–23). Although the mechanisms underlying the retardation of decomposition are not entirely clear, several studies have shown that increased N levels can suppress the activity of plant-litter-degrading enzymes, including phenol oxidases and peroxidases (19, 24, 25). In the case of ECM fungi, it is well known that increased NH_4_^+^ concentrations will reduce the biomass and alter the community composition of ECM fungi (26, 27). Less is known about how NH_4_^+^ affects the decomposition activity of ECM fungi. Laboratory-scale studies have shown that NH_4_^+^ amendments had only minute effects on the oxidation of SOM by the ECM fungus Paxillus involutus (28), whereas field studies suggest that NH_4_^+^ addition represses the oxidation-decomposing activity of ECM *Cortinarius* species (17).

In this study, we examined the effects of increased NH_4_^+^ levels on the processing of DOM, extracted by hot water from a forest soil, and the formation of mineral-associated organic C by P. involutus via two pathways: the extracellular transformation of DOM (*ex vivo* pathway) and the secretion of fungal metabolites (*in vivo* pathway) (Fig. 1). Previous experiments have shown that the decomposition of DOM by *P. involutus* is associated with the liberation and uptake of N (15). *P. involutus* assimilates N sources in a sequence, preferentially utilizing NH_4_^+^ over other N sources, such as proteins (29). Hence, our main hypothesis was that NH_4_^+^ additions will lead to decreased assimilation of organic N and thereby decreased *ex vivo* transformation of the DOM. SOM decomposition by *P. involutus* involves the action of hydroxyl radicals generated by Fenton chemistry, and secreted Fe(III)-reducing metabolites are needed to drive this reaction (30). Our second hypothesis was therefore that decreased *ex vivo* transformation will be associated with a reduced secretion of metabolites, i.e., decreased activity in the *in vivo* pathway. Since we expected that both extracellular transformation and the secretion of fungal metabolites will be negatively affected by NH_4_^+^ amendments, our third hypothesis was that the formation of mineral-associated organic C will also decrease with increasing NH_4_^+^ concentrations. In order to test these hypotheses, a firm control of the physiology of the fungus, characterization of DOM, and quantitative estimation of fungal metabolites are needed, and these were accomplished in microcosm experiments where the fungus was grown on DOM amended with different levels of NH_4_^+^. We evaluated the extracellular transformation of DOM by analyzing oxidation and depolymerization using infrared (IR) spectroscopy and size exclusion chromatography (SEC), respectively. The secretion of secondary metabolites was estimated from the production of ^13^C- and ^15^N-enriched compounds in the processed DOM after labeling the fungal mycelium with stable isotopes (^13^C and ^15^N). The formation of mineral-associated organic C was investigated by adsorption of initial and processed DOM on goethite, which is a ubiquitous soil iron mineral (31).

## RESULTS

### NH_4_^+^ treatments.

The freshly extracted DOM contained 30 mg liter^−1^ of NH_4_^+^-N, whereas NO_3_^−^ was not detected. In order to study the effects of NH_4_^+^ additions, before the experiments, a large part of the indigenous NH_4_^+^ was removed from the DOM by dialysis (cutoff, 1 kDa) (see Fig. S1 in the supplemental material). After dialysis, the NH_4_^+^-N content was 4.4 mg liter^−1^. The total N content was 25.3 mg liter^−1^; thus, organic N accounted for ca. 80% of the total N in the dialyzed DOM. NH_4_^+^-N was added at two levels to the dialyzed DOM, 15 mg liter^−1^ (denoted DOM+lowN) and 30 mg liter^−1^ (denoted DOM+highN; the concentration of NH_4_^+^-N in this medium was similar to that in the freshly extracted DOM). Two levels of NH_4_^+^-N were also added to a 2-fold-diluted DOM (dDOM) extract (i.e., 15 mg N liter^−1^ [denoted dDOM+lowN] and 42 mg N liter^−1^ [denoted dDOM+highN]) (Fig. 1B). We expected to observe a greater extent of modifications in the dDOM than in the DOM treatments. Since previous experiments have shown that the decomposition of DOM by *P. involutus* occurs only in the presence of an energy source (28), the fungus was grown for 7 days in DOM and dDOM media supplemented with glucose. Part of the added NH_4_^+^ and glucose was labeled with ^15^N and ^13^C, respectively. Amendments with NH_4_^+^ in the DOM and dDOM significantly decreased the pH of the media at the end of the incubation period (Fig. S2), in agreement with previous reports (29). The pH values dropped by 0.4 units in the medium amended with the low NH_4_^+^ level and by up to 0.7 units in dDOM+highN medium.

### Fungal biomass and C/N ratios.

**FIG 2:**
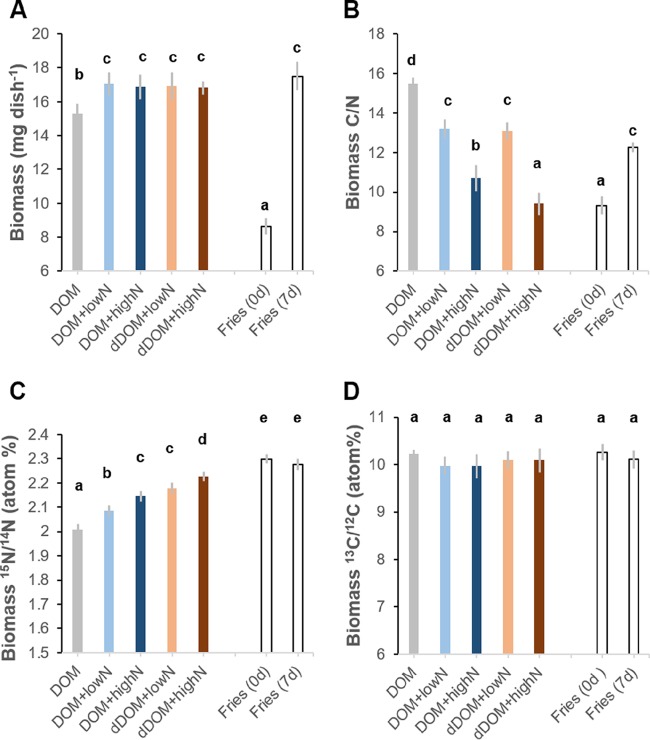
Biomass, C/N ratio, and uptake of C and N by *P. involutus* grown for 7 days on DOM or diluted DOM (dDOM) medium amended with different levels of NH_4_^+^. Data are presented as means, and error bars indicate 1 standard deviation. Open bars show the data for the fungus grown for 0 days [“Fries (0 d)”] and 7 days [“Fries (7 d)”] on Fries medium. (A) Biomass (*n *=* *10); (B) C/N ratios of the mycelium (*n *=* *5); (C) ^15^N atom% of the mycelium (*n *=* *5); (D) ^13^C atom% of the mycelium (*n *=* *5). Different lowercase letters above bars in each panel denote significant differences according to Tukey’s HSD test (*P < *0.05).

Additions of NH_4_^+^ significantly increased the biomass of *P. involutus* in both the DOM and dDOM media (*P < *0.05) (Fig. 2A; Table 1). There were no significant differences in the biomass of the mycelium grown in the DOM medium and that of the mycelium grown in dDOM medium amended with NH_4_^+^. The C/N ratio significantly decreased in the fungal mycelium that was grown in media supplemented with NH_4_^+^, suggesting increased N assimilation upon NH_4_^+^ additions. The decrease was more pronounced in the medium with the high-NH_4_^+^ amendment (*P < *0.05) (Fig. 2B).

**TABLE 1:** Effects of increased NH_4_^+^ levels on processing and formation of mineral-associated organic C during processing of dissolved organic matter by *P. involutus*^*d*^

Analysis	DOM	DOM		dDOM	
		+lowN	+highN	+lowN	+high N
Formation of mineral-associated C					
Total organic C	↑	↑	↑	(↑↑)	(↑↑)
Fungal C	↑↑↑	↑↑	↑	↑↑↑↑	↑↑

*Ex vivo* transformation^*a*^					
Depolymerization	↑	↑↑	↑↑	↑↑↑	↑↑↑↑
Oxidation	↑^*b*^	(↑)^*c*^	(↑)^*c*^	(↑)^*c*^	(↑)^*c*^

*In vivo* turnover					
N secretion	↑	↑↑	↑↑↑	↑↑	↑↑↑
C secretion	↑↑↑↑	↑↑↑	↑↑	↑↑↑	↑

Chemical composition changes of DOM^*c*^					
Organic N	↓↓↓	(↓↓)	↓	↓↓↓	(↓↓)
Organic C	↓	(↓↓)	(↓↓↓)	↓	(↓↓↓)
Reduced sugars	↓	(↓↓)	(↓↓↓)	(↓↓↓)	(↓↓↓)
Phenolics	↑↑↑	↑↑	↑	(↑↑↑↑)	↑↑

Fungal growth					
Biomass	↑	↑↑	↑↑	↑↑	↑↑

a The extent of *ex vivo* transformation during fungal processing was normalized to the total organic matter.

b Oxidation was not significant between the processed and the initial DOM (*P > *0.05).

c A change was calculated as the difference between values of the processed and the initial DOM, normalized to the value of the initial DOM.

d “↓” and “↑” denote a decrease or an increase of the measured parameter between the processed organic matter (incubated for 7 days) and the initial DOM. Within a row, the number of arrows indicates the magnitude of these changes. An arrow(s) in parentheses indicates that the values are not significantly (*P > *0.05) different comparing NH_4_^+^-amended DOM (or dDOM) and nonamended DOM that was incubated for 7 days.

### Uptake of N and C and changes in DOM composition.

**FIG 3:**
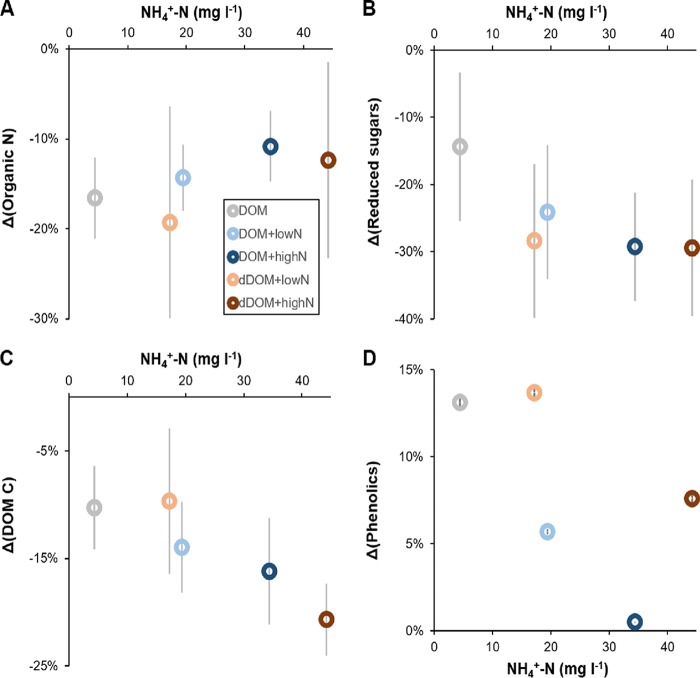
Changes in the chemical composition of the DOM processed by *P. involutus* at different NH_4_^+^ levels. Shown are changes (Δ) related to the initial contents of organic N (A), total reduced sugars (B), DOM C (C), and phenolic compounds (D). Data are represented as means (*n *≥* *4), and error bars denote 1 standard deviation. Error bars in panel D are within the symbols. Absolute values for organic N, reduced sugars, total organic C, and phenolic compounds are shown in Tables S1 and S2 in the supplemental material.

No NH_4_^+^ was detected in the media at the end of the incubation, and almost all of the added glucose was taken up by *P. involutus*, particularly in media with high-NH_4_^+^ additions (Tables S1 and S2). The chemical analysis showed that ∼10 to 20% of the organic N was taken up by the fungus (Table S1). Uptake of organic N was also inferred by isotope analysis showing that the ^15^N atom% contents of the mycelia grown on DOM and dDOM were significantly lower than that of a mycelium grown in a synthetic mineral nutrient medium spiked with an equal ^15^N atom% in added NH_4_^+^ (*P < *0.05) (Fig. 2C). There was a tendency toward high organic N uptake by the mycelium grown in the DOM medium compared to dDOM and also in the medium with the low-NH_4_^+^ amendment compared to medium with high NH_4_^+^, but these differences were not significant (*P > *0.05) (Table S1). The fraction of organic N assimilated by the fungus was slightly higher in dDOM than in DOM at similar levels of NH_4_^+^ amendment (*P > *0.05) (Fig. 3A). This is consistent with our expectation that organic compounds in dDOM were modified to a larger extent than those in DOM.

The ^13^C atom% of the mycelia grown in the DOM and dDOM media was not significantly different from that of a mycelium grown in a ^13^C-labeled mineral nutrient medium, suggesting that an undetectable amount of organic C was taken up from the DOM and dDOM media (Fig. 2D). However, the contents of both organic C and total reduced sugars decreased in the processed DOM and dDOM media compared to those in the corresponding initial media (*P < *0.05) (Fig. 3B and C; Table 1; Table S2). The decreases in DOM C and total reduced sugars tended to be larger in the DOM or dDOM medium amended with the high level of NH_4_^+^, even though these changes were not statistically significant (*P > *0.05). In contrast, the concentration of phenolic compounds in the processed media increased during incubation (*P < *0.05), and the increases were larger in the organic matter medium with low-NH_4_^+^ amendment (Fig. 3D; Table S2).

### Chemical modifications of DOM.

**FIG 4:**
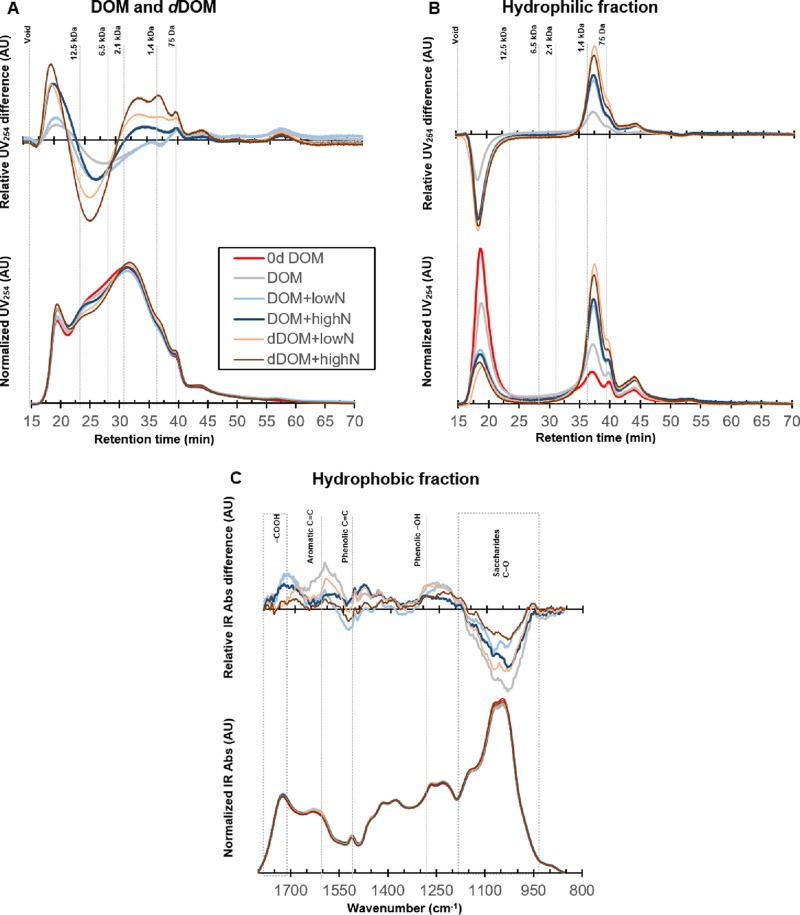
Changes in the molecular size and functional group chemistry of the DOM processed by *P. involutus* at different NH_4_^+^ levels. (A and B) Fungus-associated changes in the area-normalized size exclusion chromatograms of the processed DOM (A) and its hydrophilic fraction recovered by solid-phase extraction (B). The bottom panels present the area-normalized chromatograms, and the top panels show the differences between processed DOM and initial DOM. Note that the top and bottom panels have different scales (arbitrary units [AU]). The molecular sizes of a series of peptide standards are indicated at the top of the top panel. The elution profiles were recorded by a UV detector at 254 nm. All of the curves are shown as an average chromatogram from three repeated measurements. (C) IR spectral changes of the hydrophobic fractions of the processed DOM. (Bottom) Averages of area-normalized spectra from triplicate measurements; (top) differences between area-normalized spectra of the processed and the initial DOM. Abs, absorbance.

Size exclusion chromatography (SEC) showed that a majority of the molecules in the DOM had molecular masses of between ∼1.4 and 12.5 kDa (Fig. 4A, bottom). The changes in molecular mass distributions due to fungal processing are shown as the differences in the area-normalized SEC chromatograms between processed DOM/dDOM and the initial DOM (Fig. 4A, top), where the horizontal axis indicates no differences and values above the axis indicate increases, and vice versa. The proportion of compounds with masses of ∼2.1 to 12.5 kDa decreased after fungal processing. Concomitantly, the relative contribution of compounds of other masses (>12.5 kDa and <2.1 kDa) increased. The observed changes in molecular mass distributions were larger in the dDOM medium than in DOM, as expected. The changes were also larger in the medium with the high-NH_4_^+^ amendment (Table 1), suggesting that depolymerization was enhanced at increasing levels of NH_4_^+^.

Closer inspections of the chemical modifications of the processed organic matter were accomplished by separation into hydrophilic and hydrophobic fractions using solid-phase extraction (SPE). SEC of the hydrophilic fraction showed that the proportion of small molecules (ranging from ∼75 Da to 1.4 kDa) was higher in the processed media than in the initial organic matter (Fig. 4B). At the same time, the proportions of large molecules (>12.5 kDa) decreased, which was not observed in the SEC chromatograms of unfractionated DOM and dDOM (Fig. 4A). The observed changes in the molecular masses of organic compounds were more pronounced with the high-NH_4_^+^ amendments and also in the dDOM medium than in DOM (Fig. 4B).

Glucose remaining in the DOM/dDOM medium interfered with the IR characterization of DOM components. Therefore, we excluded glucose from the media using SPE and collected a hydrophobic fraction which contained over 80% DOM C (10). The changes in functional groups of DOM due to fungal processing are shown as the differences in the area-normalized IR spectra between processed DOM/dDOM and the initial DOM (Fig. 4C, top), where the horizontal axis indicates no differences and values above the axis indicate increases, and vice versa. Minor increases were detected in IR intensities associated with aromatic (ca. 1,600 and 1,520 cm^−1^) and phenolic (1,270 cm^−1^) functional groups in the processed DOM and dDOM compared to the initial DOM (Fig. 4C, top). These increases tended to be greater at low NH_4_^+^ levels (Fig. 4C, top). Furthermore, the intensity of the band at 1,710 cm^−1^ originating from protonated carboxyl or carbonyl functional groups was slightly increased in the processed DOM and dDOM compared to the initial DOM, indicating a higher oxidation state of C in the processed media (Fig. 4C, top). The changes were minor and were not correlated with the NH_4_^+^ levels (Table 1).

### Secretion of fungal compounds.

**FIG 5:**
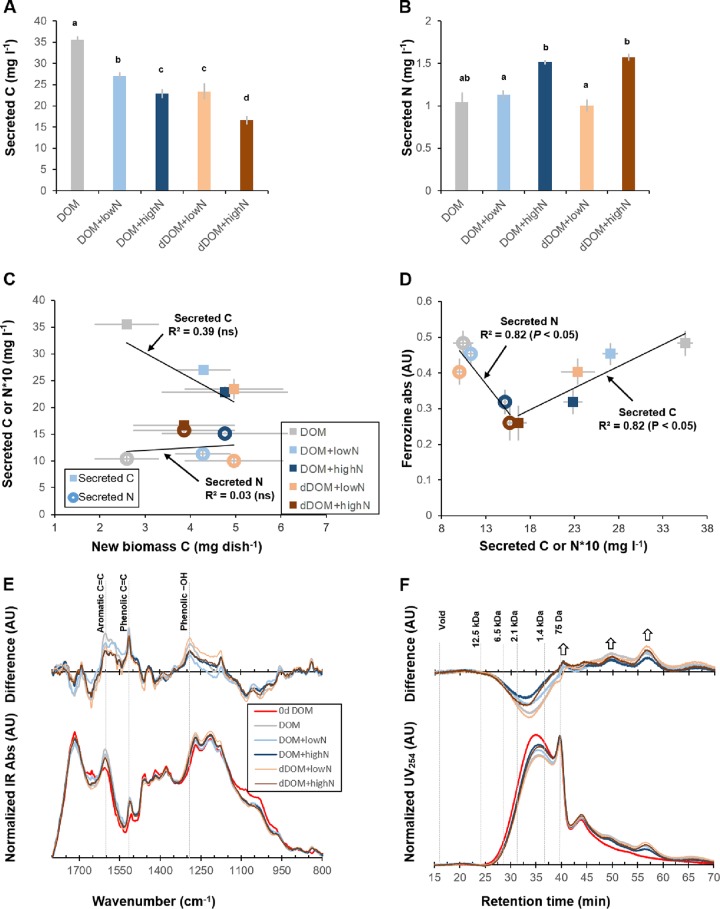
Quantification and characterization of compounds secreted into the DOM and dDOM media during processing by *P. involutus* at different NH_4_^+^ levels. Shown are the mean values ± 1 standard deviation (*n *=* *3). (A and B) Quantification of total secreted C (A) and total secreted N (B) after 7 days of fungal processing. Different lowercase letters above the bars denote significant differences according to Tukey’s HSD test (*P < *0.05). (C) Correlations between total secreted C or secreted N (for secreted N, values were multiplied by a factor of 10 for visualization) and mycelial biomass C produced during 7 days of DOM processing by *P. involutus*. ns, not significant. (D) Correlations between the amounts of secreted C or secreted N (multiplied by a factor of 10) and the levels of Fe(III)-reducing activity. (E) IR spectral changes of an ethyl acetate phase of the processed DOM. (Bottom) Area-normalized spectra of the ethyl acetate extract; (top) differences between area-normalized spectra of the processed and initial DOM. (F) Fungus-associated changes in the size exclusion chromatograms of the ethyl acetate phase of the processed DOM. The bottom and top panels present the area-normalized chromatograms and the differential chromatograms, respectively. The upward arrows indicate increased intensities likely related to fungal metabolites.

Considerable amounts of both ^13^C- and ^15^N-enriched compounds were detected in the processed DOM. The amounts of secreted compounds (in milligrams per liter of medium) were affected by the levels of added NH_4_^+^. The quantity of secreted total C decreased, whereas that of secreted total N increased, with increasing levels of NH_4_^+^ (Fig. 5A and B; Table 1). The dDOM+lowN medium had a slightly smaller amount of secreted C but a similar amount of secreted N compared to the DOM+lowN medium, indicating that fungal C secretion, but not N secretion, was influenced by the organic N concentration.

Although a previous study indicated that fungal secretion increased with fungal growth (10), we found no correlation between the amounts of secreted C and N and an increase in fungal biomass (Fig. 5C). As *P. involutus* secrets Fe(III)-reducing metabolites to drive the decomposition of DOM using Fenton chemistry (30), we related the levels of secreted C or N to the Fe(III)-reducing capacity (Fig. 5D). The concentration of secreted C, as expected, was positively correlated, whereas the concentration of secreted N was negatively correlated, with the level of Fe(III)-reducing activity detected in the processed DOM and dDOM.

Chemical characterization of the secreted metabolites was accomplished via ethyl acetate (EtOAc) extraction of the processed DOM and dDOM. This procedure has previously been used for isolating metabolites secreted by *P. involutus* during organic matter decomposition (30). The ethyl acetate fraction of the processed DOM and dDOM contained slightly higher intensities of IR bands originating from aromatic and phenolic functional groups than in the initial DOM extract (Fig. 5E). The changes were most pronounced in the fractions recovered from the media containing the highest levels of secreted C (normalized to total C in the media). These results suggest that the increase in aromatic and phenolic functional groups in the hydrophobic fraction of the processed DOM and dDOM (Fig. 4C) is partly due to the production of fungal metabolites. SEC of the ethyl acetate fraction revealed increased 254-nm-wavelength UV absorbance (UV_254_) intensities of at least three compounds after fungal processing (Fig. 5F, top). The extent of this increase was related to the increase in the amount of secreted C in the processed media.

### Formation of mineral-associated organic matter.

**FIG 6:**
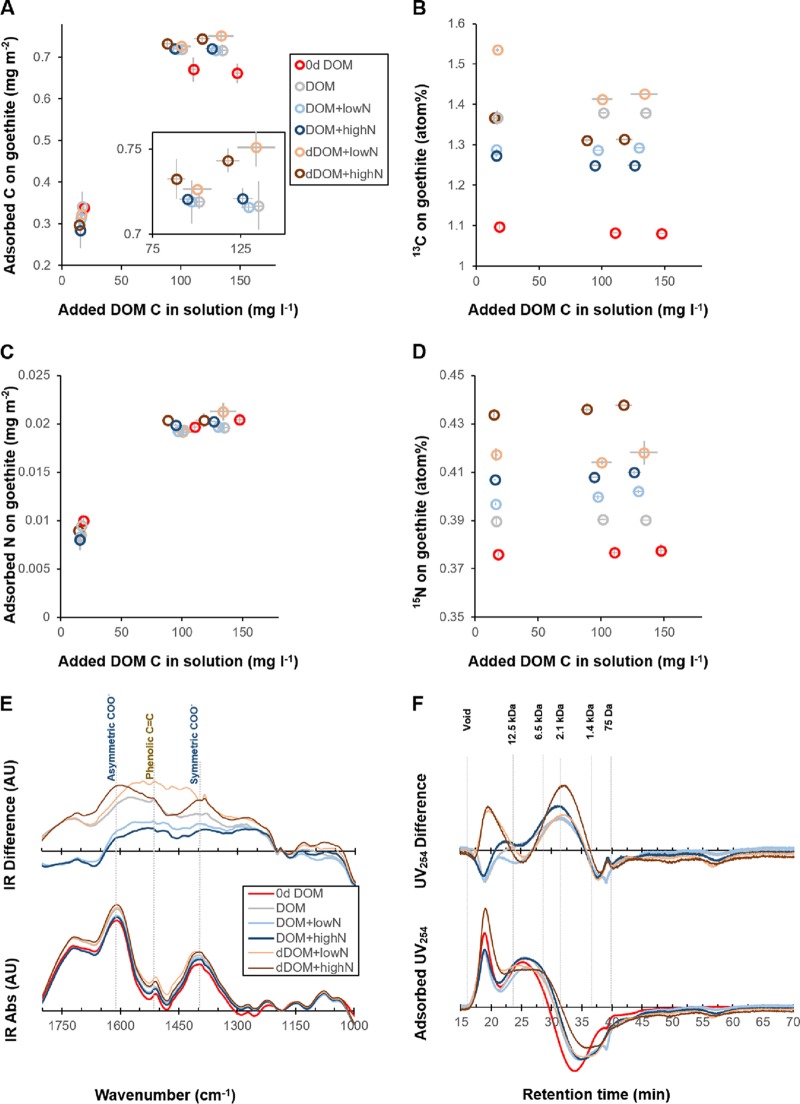
Adsorption of DOM and dDOM to goethite before and after fungal processing at different NH_4_^+^ levels. Data are presented as means, and error bars indicate 1 standard deviation (*n *=* *3, except for initial DOM [*n *=* *5], unless otherwise stated). (A) Adsorbed C on the goethite surface as a function of added DOM C. The inset shows a zoom of data points of processed DOM and dDOM at added DOM C concentrations higher than 75 mg liter^−1^. (B) ^13^C atom% of adsorbed C as a function of added DOM C. (C) Adsorbed N on the goethite surface as a function of added DOM C. (D) ^15^N atom% of adsorbed N as a function of added DOM C. (E) IR spectra of adsorbed DOM. (Bottom) Area-normalized spectra (*n *=* *2); (top) differences in spectra between the processed and initial DOM. (F) Changes in normalized size exclusion chromatograms of the adsorbed organic matter on goethite. (Bottom) Area-normalized SEC of adsorbed DOM; (top) differences in SEC chromatograms between the processed and initial organic matter. Molecular weights of a series of peptide standards are shown at the top of the top panel.

Iron oxides are omnipresent and play disproportionally important roles in stabilizing organic matter in soils (32). Therefore, we examined the reactivity of processed organic matter toward a typical iron mineral, goethite, using a batch adsorption approach. Quantitative adsorption of the initial and processed DOM and dDOM on goethite was examined in batch experiments at different concentrations of added organic C at pH 4. The adsorption of organic C was enhanced after fungal processing, especially in the dDOM medium. At the highest concentration of organic C (corresponding to complete surface saturation of DOM) (Fig. 6A; Table 1), ca. 8% to 9% more C was adsorbed in the processed DOM medium, and 12% to 14% more C was adsorbed in the processed dDOM medium, than in the initial organic matter extracts. The enhancement in adsorption was partially attributed to the secretion of fungal compounds. The adsorbed organic C in the processed DOM and dDOM samples had significantly higher ^13^C atom% than that in the initial DOM (Fig. 6B). In contrast, the adsorption of organic N onto goethite was not higher in the DOM and dDOM processed by the fungus than in the initial DOM (Fig. 6C). However, the secreted N compounds were enriched in the adsorbed fraction (Fig. 6D). According to isotope mixing models, secreted C accounted for 2 to 5% of the total adsorbed C, depending on the contribution of secreted C to total organic C (TOC) in the processed organic matter. In addition, secreted N accounted for 1 to 3% of the adsorbed N.

Approximately one-third of the observed enhancement of C adsorption was explained by fungal secretion; accordingly, the *ex vivo* transformations of indigenous organic matter by *P. involutus* contributed to two-thirds of the increased C adsorption. These transformations were further analyzed by means of IR spectroscopy of the organic matter adsorbed on goethite and SEC of the unadsorbed DOM and dDOM. The adsorbed processed DOM and dDOM possessed higher intensities of bands associated with symmetric (ca. 1,400 cm^−1^) and asymmetric (ca. 1,580 cm^−1^) carboxylate vibrations than those of the adsorbed initial DOM (33) (Fig. 6E). This indicated an increase in the average oxidation state of the adsorbed DOM and dDOM after fungal processing. The intensity was slightly higher for the DOM and dDOM media amended with high than with low NH_4_^+^ levels. Higher intensities were also detected for IR bands that originate from aromatic functional groups (1,520 and 1,600 cm^−1^) of adsorbed processed DOM and dDOM, which corroborated that compounds secreted by the fungus contributed to the adsorption of the processed organic matter. The size distribution of the adsorbed organic matter (Fig. 6F, bottom) was calculated by subtracting the area-normalized SEC chromatograms of the DOM and dDOM remaining in solution after adsorption from those of the corresponding DOM and dDOM added before adsorption. This indicated preferential adsorption of molecules with sizes larger than 2.1 kDa (Fig. 6F, bottom). The contribution of molecules, with sizes ranging from 1.4 to 6.5 kDa, to the total adsorption increased after fungal processing of both the DOM and dDOM media. The increase was greater if the media were amended with high levels of NH_4_^+^ (Fig. 6F, top). Moreover, the contribution of molecules, larger than 12.5 kDa, to the total adsorption increased in the dDOM medium after fungal processing (Fig. 6F, top).

## DISCUSSION

In agreement with data from previous experiments, the decomposition of DOM by *P. involutus* enhanced the formation of mineral-associated organic matter, partly due to the extracellular modifications of the organic matter and partly by synthesizing mineral-surface-reactive metabolites (10) (Fig. 1). We hypothesized that NH_4_^+^ amendments should lower the formation of such mineral-stabilized organic matter because increased availability of NH_4_^+^ should decrease the DOM decomposition activities that are linked to the acquisition of organic N sources. Our data showed that NH_4_^+^ amendments decreased the assimilation of organic N, but the overall production of mineral-associated organic C was not significantly affected by NH_4_^+^ additions (Table 1). However, both the *ex vivo* and the *in vivo* pathways leading to the formation of mineral-associated C were affected, but their responses to increased NH_4_^+^ levels were different. The decreased acquisition of organic N at higher NH_4_^+^ levels was not accompanied by changes in the degree of oxidation of the DOM. Instead, the extent of depolymerization increased. Moreover, increasing NH_4_^+^ levels resulted in a decreased secretion of C compounds but an increased secretion of N-containing compounds. Taken together, the observed shifts in DOM processing pathways did not increase the overall production of mineral-associated organic C, but the chemical composition of this fraction changed. Such compositional changes can potentially influence a broad range of physicochemical and biological processes in soils. These include the rates of adsorption and desorption of mineral-bound organic compounds (34), soil aggregate formation and destruction (35), the structure and activity of microbial communities, and the metabolic capacity of microorganisms (36, 37). All these processes can have profound effects on SOM stabilization (6).

The addition of NH_4_^+^ to the soil organic matter extracts significantly increased the biomass of *P. involutus* (Fig. 2A), which suggests that the fungus was N limited when grown on the dialyzed DOM extract. Amendments with higher levels of NH_4_^+^ did not further increase the mycelial biomass, indicating that under these conditions, growth was limited by nutrients and/or factors other than N. It has been shown that N from NH_4_^+^ assimilated by ECM fungi is used for biosynthesis of the amide group of glutamine or the amino group of glutamate (38). Amino acid synthesis requires a supply of C, such as glucose (39). In our experiments, more than 90% of the added glucose was utilized in the DOM and dDOM media that were supplied with low levels of NH_4_^+^, and almost all of the glucose was consumed with higher-NH_4_^+^ amendments (see Table S2 in the supplemental material). This suggests that *P. involutus* probably was limited by C at the end of incubation when supplied with high levels of NH_4_^+^. Prolonged C limitation can induce a C starvation response in *P. involutus* involving autolysis, increased secretion of NH_4_^+^, and a decline in biomass (40). Such changes in the biomass and secretion of NH_4_^+^ were not observed, which suggests that a C starvation response was not induced under any of the experimental conditions explored in this study. However, the observed decrease in the C/N ratios of the mycelial biomass grown on media with high-NH_4_^+^ amendments suggests that the metabolism of *P. involutus* was adjusted to the availability of C and N.

Recent time-resolved experiments using spectroscopy and transcriptomics have shown that the decomposition of DOM by *P. involutus* is a two-step mechanism involving oxidation and hydrolysis where oxidation precedes hydrolysis (41). Oxidation, which is a nonenzymatic mechanism involving the actions of hydroxyl radicals generated by Fenton chemistry, is initiated when NH_4_^+^ is depleted from the DOM extract and organic N sources are assimilated (29). Subsequently, when the energy source (i.e., glucose) is limited, *P. involutus* expresses a large number of hydrolytic enzymes, including proteases, chitinases, and glycoside hydrolases (41). The present study that experimentally manipulated nutritional conditions provides further support that the activities of the oxidation and hydrolytic decomposition systems in *P. involutus* are distinctively regulated in response to the availability of N and C sources. Under NH_4_^+^-limited conditions (i.e., dialyzed DOM), components in the organic matter extracts were slightly oxidized, and Fe(III)-reducing metabolites that are required for Fenton chemistry (30) were secreted. Under this condition, some depolymerization of the DOM was observed. NH_4_^+^ additions decreased the amount of secreted C compounds, which was correlated with the Fe(III)-reducing activity, and no additional oxidation of the organic matter extract was detected. In contrast, NH_4_^+^ additions increased depolymerization, likely due to increased activity in the hydrolytic pathway. Concomitantly, the concentration of secreted N compounds increased. Although the N-containing compounds were not characterized, the fact that their concentrations increased with the degree of depolymerization suggested that they contained at least some extracellular enzymes. Higher levels of NH_4_^+^ further increased the concentration of secreted N compounds, whereas the concentration of secreted C compounds decreased. Overall, our data suggest that shifts from N- to C-limited growth conditions will reduce the oxidation activity and increase the hydrolytic decomposition activities in *P. involutus* and that these changes are correlated with changes in the secretion of metabolites and enzymes. Similar shifts in hydrolase and oxidase activities were recently reported for saprotrophic fungi in a meta-analysis of soil extracellular enzyme activities under N fertilization (24).

The capacity of ECM fungi to metabolize soil C as an alternative C source to the photosynthate from the host plant remains controversial among ecologists (42, 43). Field studies using ^13^C-labeled leaf litter material in temperate forests did not indicate any incorporation of leaf litter C into the biomass (44). In contrast, other studies relying on enzyme assays of ECM root tips suggest that ECM fungi can hydrolyze SOM and that these activities are high when the supply of host C is low (45). The increased degree of depolymerization observed in our laboratory experiments with *P. involutus* was accompanied by decreased levels of organic C in the DOM extract, which suggests that at least some of the released C was taken up by the fungus. However, analysis of the mycelial ^13^C content did not show any significant levels of DOM C in the biomass (Fig. 2D). A possible explanation for this apparent discrepancy is that during low availability of energy, the assimilated C is used almost exclusively for maintenance and not for growth processes. Such nongrowing states in which viability and metabolic activity can be maintained for prolonged periods differ from starvation and have been characterized in industrially relevant fungi (46) but not yet in ECM fungi.

Recent field (47, 48) and modeling (49) studies suggest that the net effect of N additions on SOM dynamics depends on the interactions among N availability, microbial physiology, SOM decomposition, and soil minerals. In this study, we show that these interactions are complex and difficult to predict, even in a laboratory-scale microcosm with a single organism and firmly controlled nutrient conditions and DOM chemistry. We show that NH_4_^+^ amendments can influence both the *ex vivo* and *in vivo* pathways by which ECM fungi transform DOM (Fig. 1). The activity in these pathways can increase, decrease, or not change, and the processes must be considered simultaneously when investigating the net effect of N additions on the stabilization of soil C. The complex nature of these interactions may explain why the responses of SOM dynamics to N additions in the field are highly variable or even contradictory (22, 49). Our study should encourage research on more-complex soil systems to quantify the activity in the pathways underlying the microbial processing of DOM and how they affect the stabilization of SOM under N fertilization.

## MATERIALS AND METHODS

### Fungal species and culture conditions.

*P. involutus* (Batsch) Fr. (strain ATCC 200175) was maintained on modified Fries medium (50) containing 1% agar. The composition of Fries medium is d-glucose (33.3 mM or 2.5 g liter^−1^), NH_4_Cl (3.7 mM), MgSO_4_·7H_2_O (0.41 mM), KH_2_PO_4_ (0.22 mM), CaCl_2_·2H_2_O (0.18 mM), NaCl (0.34 mM), KCl (1.34 mM), H_3_BO_3_ (0.24 mM), ZnSO_4_·7H_2_O (20 μM), CuSO_4_·5H_2_O (5.01 μM), MnSO_4_·H_2_O (50.29 μM), (NH_4_)_6_Mo_7_O_24_·7H_2_O (0.16 μM), FeCl_3_·6H_2_O (73.99 μM), *myo*-inositol (55.51 μM), thiamine-HCl (0.3 μM), biotin (0.1 μM), pyridoxine (0.59 μM), riboflavin (0.27 μM), nicotinamide (0.82 μM), *p*-aminobenzoic acid (0.73 μM), and Ca-pantothenate (0.46 μM) (pH 4.8) (50). In the decomposition experiment, the fungus was grown in petri dishes on a layer of glass beads immersed in a liquid medium (51). The fungus was first grown in 10 ml of Fries medium containing [^13^C]d-glucose (ca. 10 atom% ^13^C) and [^15^N]ammonium (ca. 2.3 atom% ^15^N) for 10 days (at 18°C in the dark). At this time, the colony reached a size of approximately 4 cm in diameter and a biomass of ca. 8.5 mg (dry weight). Fries medium was removed. The mycelium and glass beads were washed with 10 ml of sterile Milli-Q (MQ) water, and 10 ml of Fries medium without N was added to induce an N-deprived mycelium (containing ca. 10 atom% [^13^C]d-glucose) (52).

After 24 h, the mycelium was washed in MQ water, and 10 ml of DOM medium was added. DOM was extracted from soil collected from the upper-10-cm soil layer of a forest site at Simlångsdalen (56°42′2.47″N, 13°6′57.75″W), Halmstad, Halland, Sweden, using hot water (53). The soil is classified as a Haplic Podzol according to the World Reference Base for Soil Resources (WRB) (54). The soil was mixed with MQ water (ratio of 1:5, wt/vol) and boiled for 1 h, and the extract was filtered through a 0.22-μm sterile PES membrane (Millipore Inc., Bedford, MA). This type of membrane was used for filtration and sterilization throughout the study if not otherwise stated. In order to remove free NH_4_^+^ and other low-molecular-weight N compounds, the DOM solution was dialyzed at 4°C against MQ water using standard regenerated cellulose dialysis tubing (cutoff of 1 kDa, Spectra/Por 7; Spectrum Laboratories Inc., Rancho Dominguez, CA). A detailed comparison between the dialyzed and undialyzed samples is presented in Fig. S1 in the supplemental material. The dialyzed DOM (denoted “DOM”) was diluted twice with an equal volume of MQ water to create a diluted DOM solution (denoted “dDOM”), which accordingly have the same C and N chemistries but different organic matter concentrations. Both the DOM and dDOM solutions were supplemented with [^13^C]d-glucose (ca. 10 atom% ^13^C) to a final concentration of 2.5 g liter^−1^. Moreover, by controlling the availability of C, the mycelium was not subjected to complete glucose depletion that would induce autolysis and an extensive release of cellular compounds from the mycelium (40). Other nutrients present in Fries medium except for N were also added. Finally, the substrates were amended with ca. 2.3 atom% [^15^N]ammonium to various N concentrations, as shown in Fig. 1B and Table S1 in the supplemental material.

The fungus was incubated in the organic matter media for 7 days. At the end of the incubation, the fungal mycelium was collected and lyophilized to determine the fungal biomass. The DOM and dDOM media were filtered (<0.22 μm) and stored at −20°C for further chemical analyses.

### Chemical analyses of fungal mycelium, DOM, and dDOM.

Total C, N, ^13^C atom%, and ^15^N atom% in freeze-dried fungal mycelium were analyzed using an elemental analysis-isotope ratio mass spectrometer (EA-IRMS) that couples an elemental analyzer (Flash 2000) via a ConFlo IV universal interface unit to a continuous-flow IRMS (Delta V Advantage; Thermo Scientific, Waltham, MA). Isotope mixing models were used to estimate the fungal uptake of C and N from the DOM (the principle of the calculation is shown below).

Media before and after fungal processing were analyzed for the concentrations of total organic C (TOC), total N, ammonium, nitrate, d-glucose, total reduced sugar and phenolics, and the specific UV absorbance at 254 nm (SUVA_254_) (55). The TOC concentration was measured using an organic C analyzer [TOC-V(CPH); Shimadzu, Kyoto, Japan]. The total N content was measured using the same apparatus equipped with a TNM-1 detector (Shimadzu, Kyoto, Japan). The ammonium and nitrate concentrations were analyzed using a flow injection analysis system (FIAstar 5000; Foss, Hillerød, Denmark). The d-glucose concentration was determined by using a glucose kit (d-glucose HK assay kit, catalog number K-GLUHK-220A; Megazyme, Wicklow, Ireland). The total reduced sugar content was analyzed by using the phenol-sulfuric acid method (56), using d-glucose as a standard. The phenolic content was determined utilizing the Folin-Ciocalteu method, as modified by Ainsworth and Gillespie (57), with tannic acid as a standard. The absorbance at 254 nm (UV_254_) was recorded using a UV-visible spectrophotometer (Ultrospec 3000; Pharmacia Biotech, Uppsala, Sweden), and the SUVA_254_ was calculated by dividing the UV_254_ by the TOC concentration (55).

To remove interferences of glucose in the spectroscopic analyses, DOM was fractionated into two fractions by solid-phase extraction (SPE) using a hydrophilic-lipophilic balanced cartridge (3-ml vac cartridge, 60 mg sorbent of a combination of the lipophilic divinylbenzene and the hydrophilic *N*-vinylpyrrolidone polymers, Oasis HLB; Waters, USA). The hydrophilic fraction, containing glucose, was not retained on the cartridge (at pH 2). The hydrophobic fraction was glucose free and collected by eluting the cartridge with 0.01 M NaOH (in a 50% methanol solution).

The DOM and dDOM in the hydrophobic fraction (acidified to pH 2) were characterized using attenuated total reflectance (ATR) Fourier transform IR (FTIR) spectroscopy (Vertex 80v; Bruker, Germany). Briefly, an aliquot of an acidified sample (10 μl) was dried under N_2_ purging to make a film on a multiple-reflection diamond crystal assembled in the MIRacle ATR accessory (Pike Technologies, Madison, WI). Each IR spectrum was recorded on the film by repeating 128 scans at a resolution of 4 cm^−1^. The background spectrum was collected under identical conditions but without the organic film. IR spectra were cut from 1,800 cm^−1^ to 850 cm^−1^ and normalized to the area below the curves.

The molecular weight distributions of DOM and dDOM (unfractionated samples) and their hydrophilic fractions from SPE (neutralized with a 0.01 M NaOH solution) were analyzed by size exclusion chromatography (SEC) using a Superdex peptide column (PC3.2/30 with an optimal separation range from 100 to 7,000 Da; GE Healthcare Life Sciences, UK) attached to a high-performance liquid chromatography (HPLC) system (Ultimate 3000; Thermo Scientific, Waltham, MA). Phosphate buffer (0.02 M phosphate and 0.25 M NaCl [pH 7.2]) was chosen as the mobile phase, with a flow rate of 50 μl min^−1^. Data were recorded by a UV detector at 214 nm, 254 nm, and 280 nm. Cytochrome *c* (molecular weight, 12.5 kDa), aprotinin (6.5 kDa), gastrin I (2.1 kDa), substance P (1.4 kDa), and glycine (75 Da) were used as molecular size standards. Each SEC chromatogram was area normalized to the total area below the curve.

### Quantification and characterization of fungal metabolites.

The labeling of fungal biomass with ^13^C and ^15^N allowed the estimation of C and N secretions during DOM processing by *P. involutus*, using isotope mixing models. The hydrophobic fraction of DOM and dDOM obtained from SPE ([^13^C]glucose free) was dried under an N_2_ stream, redissolved in MQ water, and freeze-dried for the determination of ^13^C atom% in this fraction by EA-IRMS as described above. The unfractionated processed media (no ^15^NH_4_^+^ remained after incubation) were freeze-dried for the measurement of ^15^N atom% by EA-IRMS.

The processed DOM and dDOM media contained three sources of ^13^C: secreted compounds, the remaining glucose, and DOM. In the hydrophobic DOM or dDOM fractions obtained after SPE, the glucose was removed. Therefore, the relative abundance of ^13^C (atom%) in the hydrophobic fraction (At%^13^C_medium_) could be expressed as

(1) At%C13medium=fCfungi×At%C13fungi+fCOM×At%C13NA

using the following constraint:

(2) $$1=f{\text{C}}_{\text{fungi}}+f{\text{C}}_{\text{OM}}$$

where At%^13^C_fungi_ is the abundance of ^13^C (atom%) in the fungal metabolites; At%^13^C_NA_ is the abundance of ^13^C (atom%) in the DOM or dDOM, which is equal to the natural abundance (NA) of ^13^C; and *f*C_fungi_ and *f*C_OM_ are the C fractions of the secreted metabolites and DOM/dDOM in the hydrophobic fraction, respectively.

After combining equations 1 and 2, solutions could be obtained as follows:

(3) fCfungi=At%C13medium−At%C13NAAt%C13fungi−At%C13NA

(4) fCOM=At%C13fungi−At%C13mediumAt%C13fungi−At%C13NA

Similarly, the fraction of secreted N (*f*N_fungi_) in the processed DOM/dDOM could be solved as follows, given the fact that a negligible amount of added ammonium remained after fungal processing:

(5) fNfungi=At%N15medium−At%N15NAAt%N15fungi−At%N15NA

where At%^15^N_fungi_ is the abundance of ^15^N (atom%) in the fungal metabolites, At%^15^N_medium_ is that in the processed medium, and At%^15^N_NA_ is the abundance of ^15^N (atom%) in the DOM/dDOM, which is equal to the NA of ^15^N.

The concentration of secreted C in the hydrophobic fraction of the processed DOM/dDOM (*m*C_secreted, hydrophobic_) can be estimated by equation 6:

(6) $$m{\text{C}}_{\text{secreted, hydrophobic}}=f{\text{C}}_{\text{fungi}}\times m{\text{C}}_{\text{hydrophobic}}$$

where *m*C_hydrophobic_ is the concentration of C in the hydrophobic fraction of the processed DOM/dDOM. Since SPE fractionation did not recover 100% of the secreted metabolites, a conversion factor of 0.44 was used to calculate the concentration of secreted C in the processed DOM (*m*C_secreted_) according to our previous study (10):

(7) $$m{\text{C}}_{\text{secreted}}=f{\text{C}}_{\text{fungi}}\times m{\text{C}}_{\text{hydrophobic}}/0.44$$

Likewise, the concentration of secreted N in the decomposed OM medium (*m*N_secreted_) was estimated according to

(8) $$m{\text{N}}_{\text{secreted}}=f{\text{N}}_{\text{fungi}}\times m{\text{N}}_{\text{decomposed OM}}$$

where *m*N_decomposed OM_ is the concentration of N in the processed DOM.

The Fe(III)-reducing capacity of DOM was determined using a modified ferrozine assay (58). Briefly, samples from the initial and processed DOM and dDOM media were first incubated with an equal volume of freshly prepared 1.0 mM FeCl_3_ in 0.1 M acetate buffer (pH 4.4) for 30 min. An absorbance reading was then recorded at 562 nm on a spectrophotometer after reacting with the ferrozine reagent for 1 h. The Fe(III)-reducing capacity of fungus-secreted compounds was estimated as the difference in the readings of the processed and the initial DOM and dDOM.

Metabolites were extracted from the culture filtrates using ethyl acetate (EtOAc) as described previously by F. Shah et al. (30). The EtOAc solution of the metabolites was dried under a stream of N_2_. In total, six replicate samples from the same NH_4_^+^ treatment were prepared. Three replicates were dissolved in methanol and then transferred to and dried on the ATR crystal for IR analysis. Three replicates were dissolved in MQ water and subjected to SEC.

### Adsorption of DOM on goethite.

The adsorption experiments were performed using a batch approach. Goethite was synthesized as described previously (59). The goethite had a needle shape with an estimated width of 10 to 20 nm and an estimated length of several hundred nanometers (60). The specific surface area was estimated to be 62 m^2^ g^−1^ using the N_2_ Brunauer–Emmett–Teller (BET) method (61). Three organic matter concentrations were evaluated: 0.25 ml, 1.5 ml, and 2 ml for initial and processed DOM media and 0.5 ml, 3 ml, and 4 ml for initial and processed dDOM media (see Table S2 in the supplemental material for DOM and dDOM concentrations). Each medium was mixed with 6 ml of a goethite suspension (1.17 g liter^−1^; in 0.13 M NaCl), and MQ water was added to a final volume of 10 ml. A control treatment was set up by adding 4 ml MQ water to the goethite suspension. The final adsorption suspension was adjusted to and maintained at pH 4. Adsorption was conducted for 24 h; next, the suspensions were centrifuged, and the supernatants were filtered and collected for UV_254_ and SEC analyses. After centrifugation, the goethite particles were rinsed twice with MQ water (pH 4) to remove trace amounts of loosely bound glucose (and negligible amounts of adsorbed DOM) and excess Cl^−^ ions, which otherwise may interfere with isotope analysis. One part of the goethite samples was freeze-dried for total C, total N, ^13^C atom%, and ^15^N atom% analyses using the EA-IRMS instrument (as described above). Contributions of secreted C and N to total adsorption were estimated using isotope mixing models (as described above). Another part of the goethite samples was dried, mixed with KBr powder, and analyzed using diffuse reflectance Fourier transform infrared (DRIFT-IR) spectroscopy, according to the method described by F. Rineau et al. (51). The IR spectra of adsorbed DOM were presented as the difference between spectra of the DOM-goethite complex and goethite, both of which were normalized according to the area below the goethite bands (970 to 720 cm^−1^). The molecular size distribution of adsorbed DOM was determined by the difference in area-normalized SEC chromatograms of added DOM and DOM remaining in solution after adsorption. As the initial DOM and dDOM contained identical organic C and N chemistries, we combined all initial DOM/dDOM into a group, namely, “0d DOM.”

### Data analysis.

Means were compared using one-way analysis of variance (ANOVA), using SPSS software (version 18; SPSS Inc., Chicago, IL). Tukey’s honestly significant difference (HSD) test was used to analyze the differences between groups at a significance level of a *P* value of 0.05. The data generally met the homogeneous variance assumption (tested by Levene’s test), and hence, no additional transformations were performed unless otherwise stated.

## Supplementary Material

Supplemental file 1
