# Supplementary material for: Influence of Ammonium on Formation of Mineral-Associated Organic Carbon by an Ectomycorrhizal Fungus
Source: Appl Environ Microbiol. 2019 May 2;85(10):e03007-18. doi: 10.1128/AEM.03007-18 (PMC6498167; doi:10.1128/AEM.03007-18)
Supplement: Supplemental file 1 [file AEM.03007-18-s0001.pdf]

Supplemental material

Influence of ammonium on the formation of mineral-associated organic carbon by an ectomycorrhizal fungus

Tao Wang[1, *] , Zhaomo Tian[1,2], Anders Tunlid[1] & Per Persson[1,2]

[1] Department of Biology, Microbial Ecology Group, Lund University, Ecology Building, SE-223 62 Lund, Sweden

[2] Centre for Environmental and Climate Research (CEC), Lund University, Ecology Building, SE-223 62 Lund, Sweden

[*] Correspondence and material requests to: tao.wang@biol.lu.se (Tao Wang)

SUPPLEMENTAL FIGURES

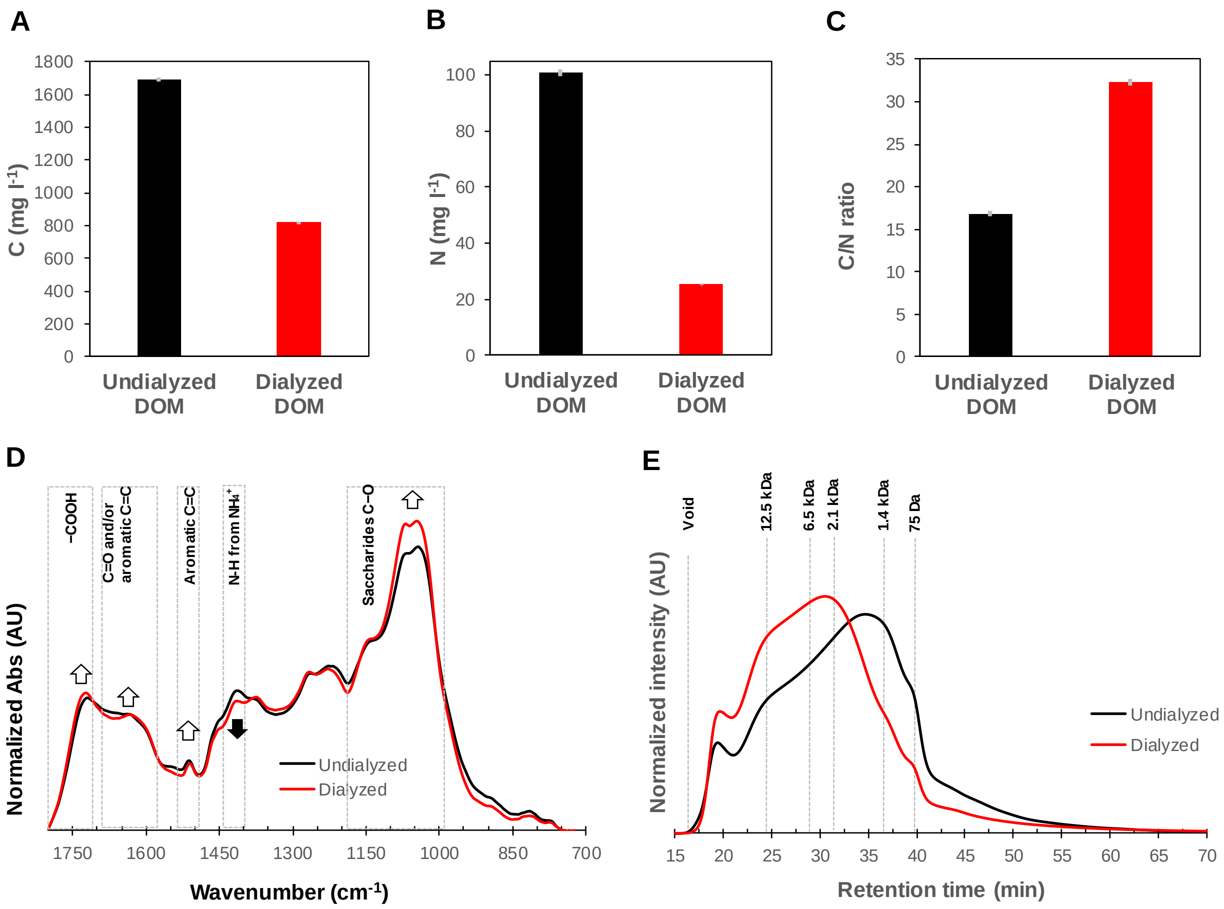

FIG S1 The effects of dialysis on selected properties of dissolved organic matter (DOM). Total C (A), total N (B), C/N ratio (C), IR spectrum (D), and area-normalized size distribution of DOM (E) changed after dialysis, analyzed using SEC at 254 nm. Data of the undialyzed and dialyzed samples are designated by black and red colors, respectively. Data are presented as means and error bars indicate one standard deviation. *n* = 3, except for IR spectra, which are the average spectrum of two replicate measurements. The downward (black) and upward arrows, in panel (D), indicate decreased and increased signals, respectively, due to dialysis. The molecular sizes of a series of peptide standards are indicated at the top of the panel (E).

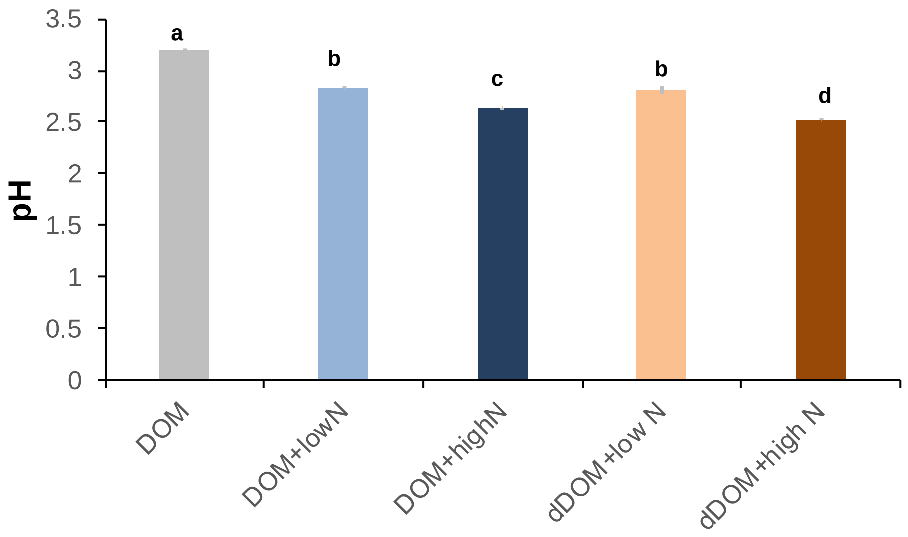

FIG S2 The pH of the DOM and dDOM media collected after the growth of *P. involutus* for 7 d at different NH_4^+ levels. Data are presented as means and error bars indicate one standard deviation (n = 3). Different lowercase letters above bars in each panel denote significant differences according to a Tukey's HSD test (P < 0.05).

SUPPLEMENTAL TABLES

TABLE S1 Contents of total N, NH_4^+-N and organic N in DOM and diluted DOM (dDOM) processed by *P. involutus* for 0 d and 7 d. Data are presented as means (in mg l^{-1}). Standard deviations are shown in parentheses, n = 2 for values at 0 d, n = 4 for values on 7 d and n = 5 for total N uptake from DOM and dDOM. Added NH_4^+-N are calculated values based on the added volume and concentration of the NH_4^+ stock solution. Values in the same row followed by different lowercase letters denote significant differences according to Tukey's HSD test (P < 0.05). Values at 0 d and NH_4^+-N uptake were not tested for differences.

	**DOM**			**dDOM**	
	**DOM**	**+lowN**	**+highN**	**+ lowN**	**+highN**
**0 d**					
**Total N**	25.3 (0.3)	37.9 (2.3)	54.2 (0.9)	27.0 (0)	53.6 (0.9)
**Added NH_4^+-N**	0	15	30	15	42
**DOM NH_4^+-N**	4.4 (0.1)	4.4 (0.1)	4.4 (0.1)	2.2 (0.1)	2.2 (0.1)
**Organic N***	20.9 (0.3)	20.9 (0.3)	20.9 (0.3)	10.5 (0.1)	10.5 (0.1)
**7 d**					
**Total N**	17.4 (0.9)a	17.8 (0.7)a	18.6 (0.8)a	8.4 (1.3)b	9.1 (1.1)b
**NH_4^+-N**	b.d. #	b.d.	b.d.	b.d.	b.d.
**N uptake from DOM and dDOM**					
**NH_4^+-N**	4.4 (0.1)	4.4 (0.1)	4.4 (0.1)	2.2 (0.1)	2.2 (0.1)
**Organic N§**	3.5 (0.9)a	3.0 (0.8)a	2.3 (0.8)a	2.0 (1.4)a	1.3 (1.1)a
**Total N¶**	6.5 (0.7)a	6.8 (0.3)a	6.3 (1.4)a	4.2 (1.2)b	2.8 (1.1)b

*Organic N was calculated as the difference between total N and NH_4^+-N.

#b.d., below the detection limit.

§Organic N uptake was estimated by subtracting organic N (equal to total N) at 7 d from organic N at 0 d.

¶Total N uptake was calculated according to the isotope mixing model using ^{15}N atom% values of fungal mycelium

.

TABLE S2 Concentrations of glucose C, DOM C, reduced sugars, phenolics and $SUVA_{254}$ of the DOM and dDOM processed by *P. involutus* for 0 d and 7 d. NH_4^+ additions did not change values in DOM and dDOM at 0 d and therefore, only one column is displayed for DOM and dDOM media at 0 d, regardless of NH_4^+ levels. Data are presented as means and standard deviations (in parentheses; $n \geq 4$). Values in the same row followed by different lowercase letters denote significant differences according to a Tukey's HSD test ($P < 0.05$).

	**DOM**				**dDOM**		
	**0 d**	**DOM 7d**	**+low N 7d**	**+highN 7d**	**0 d**	**+lowN 7d**	**+highN 7d**
**Glucose C** (mg l^{-1})	945 (35)a	224 (18)b	90 (18)c	36 (9)d	945 (35)a	75 (15)c	23 (7)d
**DOM C** (mg l^{-1})*	745 (27)a	668 (10)b	641 (16)b	624 (25)b	363 (8)c	328 (23)cd	288 (9)d
**Reduced sugars** (mg Glu eq. l^{-1}) #	1000 (71)a	856 (84)b	758 (67)bc	707 (32)c	514 (36)d	368 (45)e	363 (36)e
**Phenolics** (mg TA eq. l^{-1}) ¶	101 (1)c	114 (3)a	107 (2)b	102 (2)c	49 (1)d	56 (1)e	53 (2)de
**$SUVA_{254}$** (l (mg C m)$^{-1}$)	2.5 (0.1)ab	2.6 (0.1)a	2.5 (0.1)ab	2.5 (0.1)ab	2.5 (0.1)ab	2.4 (0)b	2.6 (0.1)ab

*Excluding contributions from glucose C.

#Glu eq., glucose equivalent.

¶TA eq., tannic acid equivalent.
